# Laparoscopic and endoscopic cooperative surgery in gastrointestinal tumors: current applications, oncologic considerations, and future directions

**DOI:** 10.3389/fonc.2026.1843117

**Published:** 2026-05-20

**Authors:** Cunyun Qiu, Haipeng Liu

**Affiliations:** 1 The Second Clinical Medical College, Lanzhou University, Lanzhou, Gansu, China; 2 Department of General Surgery, Lanzhou University Second Hospital, Lanzhou, Gansu, China

**Keywords:** Colorectal neoplasm, duodenal neoplasm, function-preserving surgery, gastrointestinal stromal tumor, gastrointestinal tumors, laparoscopic and endoscopic cooperative surgery, minimally invasive surgery, sentinel lymph node navigation

## Abstract

With the rapid development of minimally invasive gastrointestinal surgery, laparoscopic and endoscopic cooperative surgery (LECS) has emerged as a hybrid procedure that integrates the precision of endoscopic resection with the operative control of laparoscopy. By combining these complementary strengths, LECS enables complete local resection while maximizing preservation of organ function. This narrative review retrieved relevant guidelines, expert consensus statements, systematic reviews, meta-analyses, and representative clinical studies published between January 2008 and March 2026. We analyzed the technical evolution, indications, efficacy, safety, and oncological issues of LECS, and further propose a clinical application framework covering patient selection, treatment decision-making, criteria for additional radical surgery, the learning curve, structured training, and the strength of global evidence. Current evidence suggests that, in carefully selected patients, LECS can achieve high en bloc and R0 resection rates, reduce unnecessary organ resection, and facilitate postoperative recovery. However, the level of evidence varies substantially among disease entities. Relatively well-established indications include gastric gastrointestinal stromal tumors (GIST), complex colorectal polyps, and selected superficial non-ampullary duodenal tumors, whereas its role in early gastric cancer remains exploratory and is largely limited to highly selected cases involving non-exposure techniques and sentinel lymph node navigation. Overall, LECS serves as an important bridge between endoscopic therapy and conventional surgical resection. Its broader implementation, however, remains limited by oncological uncertainty in malignant tumors, a steep learning curve, inter-center heterogeneity, differences in institutional readiness, and a lack of long-term, high-quality evidence.

## 1 Introduction

The treatment paradigm for gastrointestinal tumors is shifting from an exclusive focus on radical cure toward a more balanced strategy that also prioritizes organ preservation and quality of life (1, 2). In parallel with advances in laparoscopic surgery and therapeutic endoscopy, LECS has emerged as a hybrid procedure in which surgeons and endoscopists jointly perform tumor resection and repair gastrointestinal wall defects (2–4). First introduced in Japan around 2008 for the local resection of gastrointestinal stromal tumors (2, 5), LECS has since been extended to a broader range of lesions, including early gastric cancer, colorectal neoplasms, and duodenal tumors (2–5). This expansion reflects a broader clinical need for treatments that combine oncological adequacy with functional preservation.

This expanding application has attracted increasing interest because LECS offers advantages over either modality alone. Compared with conventional laparoscopic surgery, LECS allows more accurate delineation of lesion borders under direct endoscopic visualization, thereby minimizing unnecessary resection of normal tissue (2). Compared with endoscopic resection alone, it provides laparoscopic support for full-thickness resection, defect closure, and complication management, thereby overcoming the limitations of endoscopy in perforation control and full-thickness suturing (4). These complementary advantages explain why LECS is increasingly regarded as an important bridge between endoscopic local therapy and conventional surgical resection, offering both complete excision and organ preservation when applied appropriately (1, 4).

As LECS has moved from a procedure for selected gastric submucosal tumors to a broader platform for different gastrointestinal lesions, its clinical value and limitations have become increasingly disease-specific. Against this background, a broader cross-organ evaluation is warranted. Unlike earlier reviews, which focused mainly on a single organ or the evolution of a specific technique, the present review compares the evidence across different gastrointestinal sites. Particular emphasis is placed on oncological boundaries in malignant disease, the logic of patient selection, and the challenges of standardization and dissemination. By integrating these perspectives, this review aims to provide practical guidance for clinical decision-making and future research design.

### 1.1 Literature search and review methods

**Figure 1:**
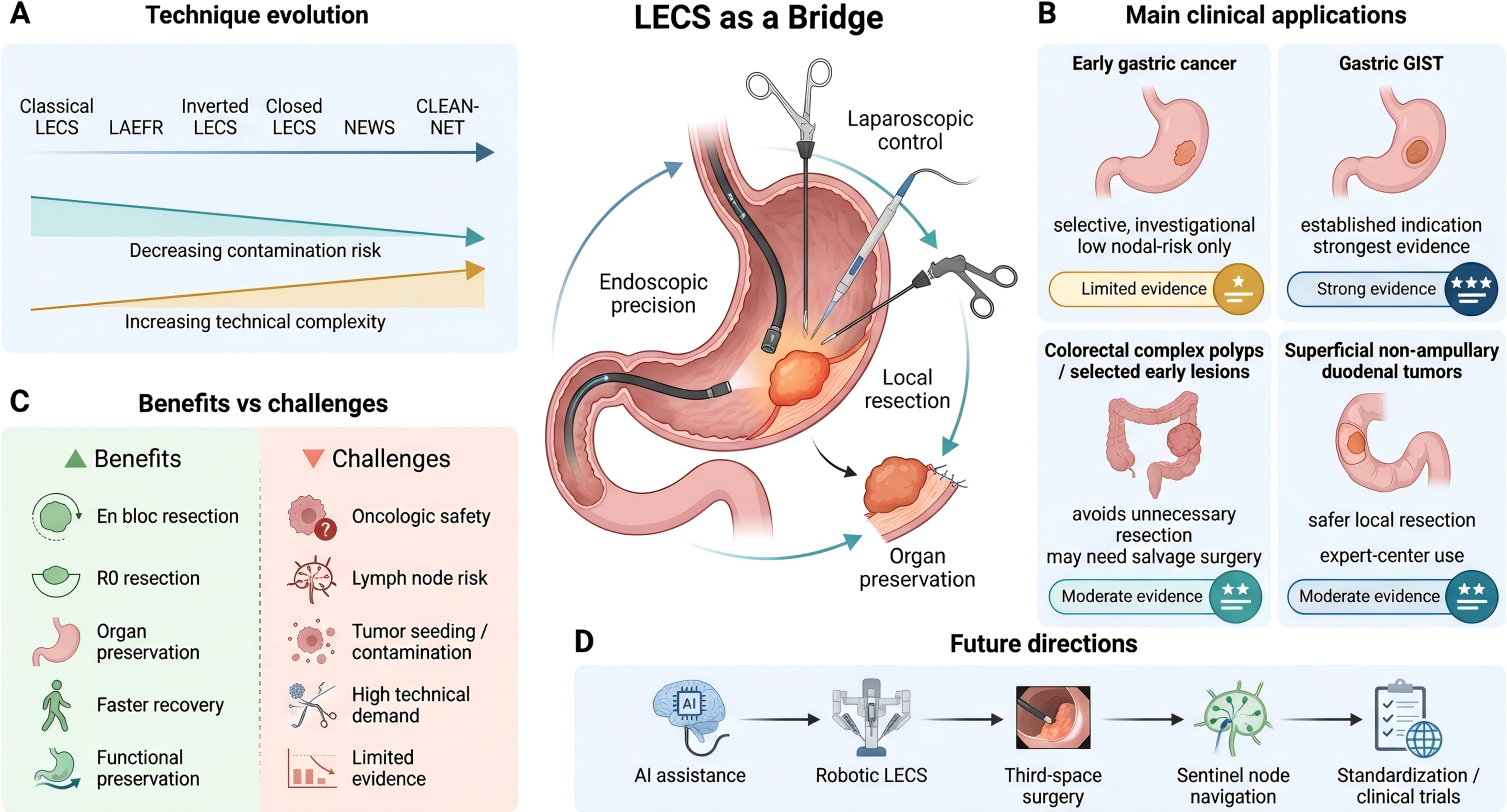
Conceptual framework of LECS in gastrointestinal tumors. In a multidisciplinary setting, LECS integrates the localization precision of endoscopy with the operative control of laparoscopy. Its principal advantages include precise local resection, organ preservation, avoidance of unnecessary tissue loss, and improved postoperative recovery. The strongest evidence currently supports its use in gastric GISTs, complex colorectal polyps, and selected superficial duodenal tumors, whereas its application in early gastric cancer remains highly selective and exploratory. Broader dissemination is limited by oncologic uncertainty in malignant disease, narrow indications, technical complexity, and dependence on specialized teams. Future progress may be driven by artificial intelligence, robotic assistance, sentinel lymph node navigation, and procedural standardization. In the image, laparoscopic instruments represent serosal- or peritoneal-side manipulation rather than routine intraluminal operation; communication between the lumen and the peritoneal cavity may occur only transiently during the defect stage after full-thickness resection.

This study is a narrative review. To enhance transparency, PubMed, Embase, and Web of Science were searched in March 2026 for literature published between January 2008 and March 2026. The search terms included “laparoscopic and endoscopic cooperative surgery,” “LECS,” “gastric cancer,” “gastrointestinal stromal tumor,” “colorectal tumor/polyp,” “duodenal neoplasm,” “sentinel node,” and “non-exposure technique.” Priority was given to guidelines, expert consensus statements, systematic reviews, meta-analyses, prospective studies, and representative multicenter or key single-center studies published within the previous 5 years. Seminal reports describing foundational procedures were also retained to clarify the historical and technical development of LECS. Conference abstracts, duplicate publications, and studies with extremely small sample sizes that could not support key conclusions were not considered primary evidence sources. Because this was a narrative rather than a systematic review, no formal quantitative assessment of risk of bias was performed. Nevertheless, where possible, the discussion distinguishes among evidence derived from guidelines, meta-analyses, prospective studies, and retrospective studies. **Figure 1** summarizes the conceptual framework of LECS in gastrointestinal tumors.

To facilitate comparison of the global evidence base across different indications, this review qualitatively stratifies the strength of relevant evidence according to study design, sample size, multicenter or prospective status, follow-up duration, consistency of outcome measures, and external reproducibility. This stratification does not constitute a formal GRADE assessment. Instead, it is intended to improve the clinical readability of this narrative review. Evidence derived from multicenter studies, systematic reviews, or meta-analyses with consistent outcomes is classified as “relatively strong” or “moderate,” whereas evidence mainly originating from small-sample, single-center, or indirect data is classified as “limited” or “exploratory.” This approach allows the evidence for each indication to be interpreted within its methodological context.

On the basis of this search strategy and evidence framework, the following sections first summarize the theoretical basis and technical evolution of LECS, then examine its disease-specific applications, current challenges, and future directions. The major procedural categories and disease-specific evidence are summarized in **Tables 1**, **2**.

**Table 1:** Classification and technical characteristics of LECS procedures.

Procedure	Exposure status	Main technical principle	Typical indications	Main advantages	Main limitations
Classical LECS	Exposure	Endoscopic marking with laparoscopic full-thickness resection	Gastric submucosal tumors	Precise margin definition	Risk of peritoneal contamination or tumor exposure
Inverted LECS	Reduced exposure	Inversion of lesion before resection	Selected gastric lesions	Less peritoneal contamination	Technically demanding
Closed LECS	Non-exposure	full-thickness resection without lumen opening	Early gastric neoplasms	No peritoneal spillage	limited indications
NEWS	Non-exposure	Non-exposed wall-inversion technique	Small gastric lesions	No contamination	Size limitation
CLEAN-NET	Non-exposure	Laparoscopic resection without luminal breach	Gastric submucosal tumors	Stapled closure	Less precise margins
LAEFR	Exposure	endoscopic full-thickness resection assisted by laparoscopic monitoring and closure	Difficult gastrointestinal lesions	Expanded closure	Potential contamination risk; technically demanding
D-LECS	Hybrid	Laparoscopic-assisted duodenal tumor resection	superficial non-ampullary duodenal tumors	Expanded resectability	Narrow indications
Third-space or robotic LECS	Variable	Third-space or robotic-assisted approaches	Selected cases	Enhanced capabilities	LECS-specific evidence remains limited

**Table 2:** Representative evidence and clinical positioning of LECS in different gastrointestinal tumors.

Tumor type	Main selection criteria	Key tumor characteristics	Common LECS strategy	Key outcomes (representative data)	Major complications/concerns	Follow-up and recurrence	Current role
Early gastric cancer	Primarily cT1a disease; lesions at the boundary of the expanded indications for ESD or technically difficult to resect endoscopically; a clear need for function-preserving treatment; preferably combined with sentinel-node navigation.	Superficial epithelial lesions; low risk of lymph-node metastasis required; obvious deep invasion or lymph-node metastasis should be excluded.	Non-exposure techniques such as NEWS or closed LECS combined with sentinel-node biopsy/OSNA.	Evidence is mainly derived from small case series; in a pilot study of NEWS plus OSNA, local resection was successfully completed in all 5 patients, with no sentinel-node metastasis detected.	Peritoneal dissemination, missed lymph-node metastasis, and possible need for additional radical surgery.	Long-term evidence demonstrating oncological equivalence remains insufficient.	Investigational; only for highly selected cases.
Gastric GIST	Resectable localized gastric GIST, especially tumors adjacent to the cardia or pylorus, or cases requiring maximal gastric preservation.	Mostly small- to medium-sized submucosal tumors; routine lymph-node dissection is generally unnecessary.	Conventional LECS, NEWS, CLEAN-NET, and closed LECS.	In a multicenter series of 126 patients, conversion to open surgery was 1.6%, complications occurred in 4.8%, and no local or distant recurrence was observed during a median follow-up of 54.7 months; in a single-center series of 100 patients, the R0 resection rate was 100%.	Tumor rupture, pseudocapsule injury, and luminal stenosis or deformity.	The most mature medium- to long-term outcomes are available; recurrence rates are low.	Relatively established indication.
Colorectal tumors/polyps	Endoscopically challenging but localized lesions; complex large polyps, non-lifting lesions, and lesions involving the appendiceal orifice, adjacent to a diverticulum, or located at the ileocecal valve.	Predominantly benign lesions or low-risk early cancers; invasive carcinoma can be difficult to exclude completely before surgery.	ESD/EMR combined with laparoscopic assistance for localization, traction, and local full-thickness or wedge resection.	A systematic review of 1,112 cases reported additional surgery in 5%, complications in 7%, reoperation in 1%, and local recurrence in 3%; a meta-analysis of 715 cases showed shorter hospital stay and fewer complications compared with segmental colectomy.	Pathological upgrading after surgery, need for additional radical resection, and a substantial learning curve.	Overall recurrence is low, but subsequent surgery should be determined according to pathological risk stratification.	Important complementary approach.
Duodenal tumors	Superficial non-ampullary lesions; local resection is feasible; no clear evidence of deep invasion or lymph-node metastasis.	Adenomas, intramucosal carcinomas, and selected neuroendocrine tumors; anatomically high-risk location.	D-LECS, consisting of endoscopic resection/dissection plus laparoscopic suturing and reinforcement.	A multicenter series of 206 patients reported an en bloc resection rate of 96.1%, an R0 resection rate of 95.1%, and no recurrence; a 2025 single-center series of 18 patients reported no recurrence at a median follow-up of 37 months.	Delayed perforation, leakage, prolonged operative time, and strong center dependence.	Medium- to long-term data are accumulating, but the quality of evidence remains limited.	Promising in expert centers.

## 2 Theoretical basis and technical evolution of LECS

### 2.1 Technical principles

The central concept of LECS is the integration of laparoscopy and gastrointestinal endoscopy within a single operative workflow (6). Within a multidisciplinary framework, this integration enables coordinated lesion resection and defect management. During the procedure, laparoscopy establishes the intra-abdominal operative field and provides serosal traction and suturing capability, whereas endoscopy enables intraluminal localization, mucosal and submucosal dissection, and precise margin assessment (6, 7). Through this cooperation, LECS achieves a refined balance among the extent of local resection, the route of full-thickness incision, and the method of defect closure.

In classical LECS, the lesion is first marked endoscopically, followed by circumferential mucosal and submucosal incision. Laparoscopic full-thickness transection is then performed from the serosal side. After resection, the defect is closed laparoscopically, and endoscopy is used to confirm closure integrity and luminal patency. This sequence illustrates the core rationale of LECS: endoscopy guides precise resection, while laparoscopy ensures secure full-thickness closure and intra-abdominal safety. By combining these functions, LECS enables minimally invasive local excision of lesions that previously would have required open surgery or more extensive resection (6).

### 2.2 Technical evolution and classification

Building on these basic principles, LECS has evolved as its limitations have become increasingly recognized. With a better understanding of the biological heterogeneity of gastrointestinal tumors and increasing concern regarding peritoneal contamination and tumor dissemination, several modified LECS procedures have been developed (1, 7). Classical LECS was initially applied mainly to benign lesions or lesions with low malignant potential arising from the gastric wall, such as gastric GISTs. However, because this approach temporarily exposes the gastrointestinal lumen during full-thickness resection, it is generally unsuitable for lesions with malignant mucosal components (3, 7).

To reduce the risks of digestive fluid leakage and peritoneal tumor-cell dissemination, several non-exposure or low-exposure techniques were subsequently developed (7, 8). Broadly, two developmental directions emerged: modified forms of classical LECS that reduce luminal exposure, and fully non-exposure approaches designed to prevent direct communication between the lesion and the peritoneal cavity. Modified LECS, also known as inverted LECS, the crown method, or flipped LECS, uses circumferential seromuscular incision and traction sutures to invert the lesion into the lumen before endoscopic full-thickness resection. In closed LECS, circumferential submucosal incision is first performed endoscopically, followed by serosal marking and inversion suturing. Endoscopic full-thickness resection is then completed without direct exposure of the tumor to the peritoneal cavity (8, 9). Thus, these approaches retain the precision of endoscopic resection while reducing the oncological and infectious risks associated with luminal exposure.

Several more specialized procedures have also been developed. Non-exposed endoscopic wall-inversion surgery (NEWS) preserves mucosal integrity while laparoscopic circumferential seromuscular incision is performed and the lesion is inverted into the lumen before full-thickness resection. Combination of laparoscopic and endoscopic approaches to neoplasia with non-exposure technique (CLEAN-NET) achieves local wedge resection mainly from the serosal side while attempting to preserve the mucosal barrier (8, 9). Other variants have been designed for specific anatomical locations or specimen-retrieval needs, including laparoscopy-assisted endoscopic full-thickness resection (LAEFR), closed endoscopic full-thickness resection, and duodenal LECS (D-LECS) (10, 11). Overall, LECS-related procedures can be categorized into exposure and non-exposure types. Non-exposure procedures offer greater protection against peritoneal contamination but require more advanced technical expertise (7, 8, 11).

### 2.3 The key role of multidisciplinary collaboration

Successful implementation of LECS depends not only on procedural refinement but also on close collaboration among surgeons, endoscopists, anesthesiologists, pathologists, and radiologists (6, 12). Preoperative evaluation with endoscopy, endoscopic ultrasonography, and imaging helps identify patients suitable for local resection. Once an appropriate patient is selected, real-time intraoperative communication is essential to ensure safety and precision. For example, in D-LECS, laparoscopic serosal suturing combined with continuous endoscopic monitoring enables prompt detection and management of perforations that would be difficult to control endoscopically alone (13, 14). Similarly, in colorectal LECS, intraoperative colonoscopy facilitates localization of occult lesions and assessment of resection margins (15, 16).

More broadly, LECS has promoted deeper integration between surgery and endoscopy and has introduced a more individualized treatment model for gastrointestinal tumors. Its technical principles, evolutionary trajectory, and multidisciplinary framework provide the basis for its expanding application across tumor types. These technical and organizational foundations also underlie the disease-specific applications discussed below (12).

## 3 Clinical applications of LECS in gastrointestinal tumors

Building on these technical foundations, LECS has gradually been applied to a range of gastrointestinal tumors. Its clinical role differs among disease types because of marked differences in biological behavior, risk of lymph node metastasis, anatomical location, and the value of local resection. To facilitate comparison across tumor types, the following sections examine representative evidence in terms of patient selection, tumor characteristics, procedural strategy, key outcomes, and current clinical positioning. The discussion proceeds from the most oncologically controversial setting to more established indications and then to anatomically high-risk applications.

Before discussing individual indications, it is important to clarify a general decision-making framework. In clinical practice, decision-making for LECS should follow the principle of “first determining the biological risk of the tumor, then assessing the value of local resection, and finally evaluating technical and institutional feasibility (17).” The proposed workflow is as follows. Preoperative assessment should integrate white-light and magnifying endoscopy, endoscopic ultrasonography, CT/MRI, and pathological biopsy to determine lesion depth, extent, and lymph node risk (17–19). If the lesion can be safely treated by endoscopic mucosal resection (EMR), endoscopic submucosal dissection (ESD), or endoscopic full-thickness resection (EFTR), and if the defect can be closed endoscopically, endoscopic treatment alone should be prioritized (18, 19). Conversely, if definite lymph node metastasis, high-risk invasive carcinoma, or the need for systematic lymphadenectomy is present, standard radical surgery should be preferred (20). Between these two scenarios, LECS may be considered when the lesion is localized and the value of local resection is clear, but endoscopic resection alone carries a high risk or laparoscopy alone cannot adequately balance precise localization with functional preservation (17). For mucosal malignant or suspiciously malignant lesions, non-exposure or low-exposure techniques should be prioritized (17, 18). Sentinel node navigation, intraoperative frozen-section pathology, or postoperative pathological stratification should also be incorporated to determine whether additional radical treatment is required (20–22).

### 3.1 Early gastric cancer

#### 3.1.1 Procedure and indications

Among epithelial gastric malignancies, early gastric cancer is the most controversial application of LECS and therefore requires the greatest caution. Standard treatment remains ESD, EMR, or radical gastrectomy with appropriate lymph node dissection. Against this standard, the potential value of LECS lies mainly in offering a function-preserving strategy that combines local resection with nodal evaluation for a very limited subset of patients. These patients typically have lesions at the margin of expanded ESD indications, lesions that are technically difficult to resect endoscopically, or lesions for which maximal preservation of gastric volume and function would provide clear benefit. Even in such cases, this strategy is justifiable only when the risk of lymph node metastasis is rigorously controlled (17, 23, 24).

Because classical LECS creates communication between the gastric lumen and the peritoneal cavity during resection, it is generally unsuitable for malignant mucosal lesions. Current exploration has therefore focused on non-exposure procedures, such as NEWS and closed LECS, combined with sentinel node biopsy or one-step nucleic acid amplification (OSNA) (17, 25, 26). These combined strategies are intended to address two major oncological concerns: peritoneal dissemination and occult nodal metastasis. Although prospective Japanese studies have demonstrated high detection rates and diagnostic accuracy for sentinel node navigation in early gastric cancer, these findings do not establish LECS as a standard treatment (25, 27).

#### 3.1.2 Efficacy and safety

Given these strict indications, direct evidence supporting LECS for gastric cancer remains limited to small case series and exploratory studies. In a preliminary study by Crafa et al. (26), NEWS combined with one-step nucleic acid amplification was performed in only five patients, all of whom underwent successful local resection without detectable sentinel node metastasis. Although these findings support technical feasibility, the sample size is too small to justify broad clinical adoption.

Higher-level evidence is available for stomach-preserving surgery guided by sentinel node navigation rather than for LECS itself. The long-term results of the SENORITA trial showed that sentinel node navigation-based stomach-preserving surgery improved quality of life and nutritional outcomes. However, it did not demonstrate non-inferiority to standard surgery in terms of 3-year disease-free survival. Taken together, these findings support further exploration of function-preserving treatment in highly selected patients, rather than replacement of standard gastrectomy (21, 28, 29).

Beyond the limited efficacy data, oncological safety remains the central concern in malignant settings. Peritoneal contamination and implantation metastasis represent the most important risks. Ohki et al. (30) reported that, when the gastric lumen was opened, the method of lavage influenced the detection of free cancer cells in washing fluid: the detection rate was 58% with Ringer’s solution and 6% after distilled water lavage. Although these findings cannot be directly translated into clinical recurrence risk, they support avoidance of exposure-type LECS in epithelial malignancies and emphasize the importance of non-exposure techniques and protective lavage strategies (17, 30).

The appropriateness of local resection also depends on reliable assessment of lymph node metastatic risk. Studies of undifferentiated early gastric cancer have shown that lymph node metastasis occurs in approximately 5.9% of intramucosal cancers and may increase to 29.2% when submucosal invasion is present (31). The risk is even higher in tumors measuring at least 2 cm or showing lymphatic invasion. Thus, the key prerequisite for LECS in early gastric cancer is not simply small tumor size, but a sufficiently low and intraoperatively verifiable risk of nodal metastasis (23–25, 31).

#### 3.1.3 Controversies and prospects

Overall, LECS in early gastric cancer should still be considered an exploratory adjunct rather than a standard option. Appropriate candidates are likely limited to patients with very early-stage disease, low risk of lymph node metastasis, a clear expected benefit from function preservation, and access to non-exposure procedures and sentinel node navigation in highly experienced centers (17, 24, 32, 33).

Accordingly, LECS cannot currently replace standard gastric cancer surgery. At most, it may serve as one component of a function-preserving treatment pathway under strict patient selection, multidisciplinary collaboration, and fully informed consent. This positioning is particularly important because local resection remains conditional on both intraoperative assessment and final pathology (17, 25, 28, 33).

For early gastric cancer, if postoperative pathology after LECS indicates that the criteria for curative endoscopic or local resection are not met, timely conversion to, or additional performance of, radical gastrectomy with appropriate lymph node dissection should be undertaken (18, 20, 34). Specific indications include a positive or unevaluable vertical margin, deep submucosal invasion, lymphatic or vascular invasion, poorly differentiated or undifferentiated components beyond the safe range, tumor diameter or ulcerative status exceeding low-risk criteria, positive sentinel nodes or one-step nucleic acid amplification results, and suspicious lymph nodes detected on imaging or intraoperatively (18, 20, 21, 34, 35). If an intraoperative non-exposure strategy fails, resulting in tumor exposure or evident intraperitoneal contamination, the threshold for conversion to standard radical surgery should also be lowered (17, 20).

Having outlined the most oncologically controversial application, the discussion now turns to colorectal tumors, where the primary clinical value of LECS lies less in replacing radical cancer surgery than in avoiding unnecessary bowel resection for complex lesions.

### 3.2 Colorectal tumors

#### 3.2.1 Procedure and indications

In colorectal disease, LECS is used primarily to avoid overtreatment of benign lesions or lesions confined to the mucosa or superficial submucosa (12, 36). With the expansion of colorectal screening, clinicians increasingly encounter complex polyps that are large, broad-based, fibrotic, or non-lifting, or that arise in anatomically challenging locations such as the ileocecal valve, appendiceal orifice, or areas adjacent to diverticula (36–40). Traditional management often requires segmental bowel resection. However, pooled evidence suggests that only about 12% of lesions treated by hybrid laparoscopic-endoscopic local excision are ultimately confirmed to be adenocarcinomas (12). This finding implies that many patients might otherwise undergo unnecessary bowel resection.

In colorectal LECS, laparoscopy typically provides serosal exposure, traction, or lesion localization, whereas endoscopy is used for polypectomy or submucosal dissection. If endoscopic resection is incomplete, laparoscopic wedge resection can be added. If intraoperative findings indicate invasion beyond the limits of local resection, the procedure can be converted immediately to standard oncological surgery (12, 41). This flexibility makes colorectal LECS particularly attractive as a “local-first, escalate-if-needed” strategy.

#### 3.2.2 Efficacy and safety

Recent evidence has strengthened support for the short-term benefits of colorectal LECS. In a systematic review of 1,112 complex colorectal lesions, Distefano et al. (12) reported that only 5% of patients required additional surgery, 7% underwent radical resection because final pathology showed malignancy, the overall complication rate was 7%, the reoperation rate was 1%, and the local recurrence rate was 3%. More importantly, a 2025 meta-analysis of 13 studies involving 715 patients found that, compared with laparoscopic segmental resection, LECS significantly reduced hospital stay, operative time, blood loss, and overall postoperative complications (42). These findings suggest that, in appropriately selected patients, LECS not only preserves bowel but may also improve perioperative recovery.

Earlier representative series also support its feasibility. Tamegai et al. (4) reported successful completion of all 17 LECS-CR procedures without conversion to open surgery, with an R0 resection rate of 100%, no anastomotic leakage or stenosis, and no local residual disease or recurrence over a median follow-up of 30.8 months. Similarly, a 10-year cohort study by Golda et al. (43) showed that LECS reduced hospital stay and complications while sparing many patients with benign lesions from planned segmental colectomy. Together, these data support the role of colorectal LECS as a bowel-preserving option for carefully selected complex lesions.

#### 3.2.3 Controversies and patient management

Despite these encouraging results, colorectal LECS should be performed only after strict patient selection, and its postoperative management should follow a two-stage strategy driven by final pathology (42, 44). Because invasive carcinoma cannot be completely excluded before local resection, the need for additional radical bowel resection with lymph node dissection should be assessed when final pathology reveals high-risk features, such as deep submucosal invasion, lymphovascular invasion, poor differentiation, high-grade tumor budding, a positive or unevaluable vertical margin, unreliable pathological staging due to tumor fragmentation, or radiological or intraoperative findings suggestive of regional lymph node metastasis (39, 44–46). It should be noted that these escalation criteria are not derived from LECS-specific prospective trials, but are mainly extrapolated from established treatment principles for T1 colorectal cancer and malignant colorectal polyps (39, 44–46). Therefore, colorectal LECS is better understood as an organ-preserving therapeutic platform characterized by “local resection first, pathology-guided escalation when indicated,” rather than as a definitive one-step solution for all colorectal tumors (12, 42).

Outcomes are also highly sensitive to lesion location, fibrosis, resection method, and institutional experience. The learning curve is substantial, and inter-center heterogeneity is considerable (12, 42, 47). Accordingly, the best indications are likely complex benign polyps, endoscopically difficult lesions, and very low-risk early cancers, rather than all suspicious malignant colorectal lesions.

Compared with early gastric cancer and colorectal epithelial lesions, gastric GIST provides a more favorable oncological context for LECS because lymph node dissection is rarely required and function-preserving local resection is often oncologically appropriate.

### 3.3 Gastrointestinal stromal tumors

#### 3.3.1 Procedure and indications

Compared with epithelial malignancies, GIST represents one of the most mature indications for LECS and offers the clearest oncological rationale. Current guidance from the Japan Society of Clinical Oncology (JSCO) and the National Comprehensive Cancer Network (NCCN) consistently emphasizes that the surgical goals for resectable localized GIST are complete R0 resection and avoidance of tumor rupture. Routine lymph node dissection is usually unnecessary, and organ- or function-preserving local resection is preferred whenever oncological safety can be maintained (48–50). This principle aligns closely with the core objective of LECS: precise local excision.

For lesions near the cardia, pylorus, or esophagogastric junction, conventional laparoscopic wedge resection may remove excessive normal gastric wall and cause luminal deformity. With endoscopic guidance, LECS allows more accurate definition of lesion margins and the incision line, thereby maximizing preservation of the normal gastric wall and gastric emptying function (2, 51). For lesions with mucosal ulceration or concern regarding intraluminal contamination or exposure, non-exposure or low-exposure procedures such as NEWS, CLEAN-NET, and closed LECS may be more appropriate. Thus, the choice of LECS subtype should be guided by tumor location, growth pattern, and exposure risk (2, 7, 8).

#### 3.3.2 Efficacy and safety

Among all LECS indications, gastric GIST currently has one of the strongest evidence bases. Recent multicenter studies have shown that classical LECS and its modified variants for gastric submucosal tumors achieve favorable short- to mid-term outcomes, including high R0 resection rates and low perioperative complication rates (52). In an earlier multicenter study of 126 cases from eight institutions, 68.3% of lesions were GISTs, the conversion rate to open surgery was only 1.6%, the complication rate was 4.8%, and no local or distant recurrence was observed over a median follow-up of 54.7 months (52, 53). A subsequent study involving 21 Japanese institutions further supported the overall safety of the technique (52).

A single-center series of 100 cases likewise demonstrated complete resection with negative margins, rapid resumption of oral intake, and few major complications (54). Compared with simple wedge resection, LECS and procedures such as NEWS appear better able to minimize unnecessary resection of normal gastric wall, especially when both R0 resection and preservation of function are priorities (55). Thus, GIST is not merely a feasible indication for LECS; it is currently the clearest example of the technique’s unique clinical value.

It should be emphasized that “treatment escalation” in the context of GIST does not equate to routine lymph node dissection (50, 56, 57). Instead, it should be centered on tumor integrity and risk stratification (48, 50, 56, 57). If tumor rupture, pseudocapsule injury, a positive margin, or suspected local residual disease occurs during LECS, the need for repeat local resection, extended resection, close surveillance, and adjuvant targeted therapy should be evaluated through multidisciplinary discussion (48, 50, 57–59). For high-risk or ruptured cases, short-term R0 resection should not be simplistically equated with long-term oncological safety (48, 56, 58, 59).

#### 3.3.3 Application of innovative technologies

Because the oncological boundaries of local treatment are comparatively well defined in GIST, and because function-preserving local excision is already a legitimate goal, this disease has emerged as an important platform for technological innovation, particularly in robotics and third-space surgery. Third-space robotic and endoscopic cooperative surgery (TS-RECS) uses robotic assistance to dissect within the submucosal “third space,” preserving the gastric mucosal layer as much as possible while ensuring complete tumor removal. In a prospective study of 20 patients with gastric GIST, the mean tumor diameter was 33.0 mm, the R0 resection rate was 100%, mucosal integrity was preserved in 95% of cases, no major adverse events occurred, and the median hospital stay was 6 days (60).

A propensity score-matched study further showed that, compared with laparoscopic wedge resection, TS-RECS was associated with less blood loss, earlier oral intake, shorter hospitalization, and better postoperative gastrointestinal function scores, without inferior short-term oncological outcomes (60, 61). In addition, a longitudinal nested cohort study from China comparing robotic-endoscopic cooperative surgery (RECS), laparoscopic surgery, and open surgery found that RECS reduced intraoperative bleeding, accelerated bowel recovery, alleviated postoperative gastrointestinal symptoms, and shortened hospital stay. Although the overall cost was higher, cost-effectiveness analysis suggested a high probability of cost-effectiveness across multiple willingness-to-pay thresholds (62). Taken together, these findings suggest that robotic platforms may further expand the function-preserving potential of LECS, although broader validation is still required.

While gastric GIST illustrates the value of LECS in a biologically favorable setting, duodenal tumors highlight its potential role in anatomically complex and high-risk locations where endoscopic resection and conventional surgery both have important limitations.

### 3.4 Duodenal tumors

#### 3.4.1 Procedure and indications

If GIST represents a relatively mature indication for LECS, duodenal tumors illustrate its unique value in anatomically high-risk settings (63–65). Non-ampullary duodenal tumors include adenomas, intramucosal adenocarcinomas, and neuroendocrine tumors. Because the duodenal wall is thin, richly vascularized, and anatomically complex, endoscopic resection alone is technically demanding and carries a substantial risk of complications. Conventional surgery, however, is often more invasive (64–67). This clinical dilemma provides the rationale for D-LECS.

D-LECS was developed to combine the precision of endoscopic resection with the safety of laparoscopic monitoring and repair. The typical workflow includes endoscopic mucosal incision or submucosal dissection of the lesion, combined with simultaneous laparoscopic monitoring of the serosal surface and immediate repair or reinforcement when perforation or a high-risk defect occurs (63–66). For lesions on the lateral duodenal wall, either an antecolic or transmesocolic approach may be selected according to tumor location to optimize exposure. At present, D-LECS is primarily applied to superficial non-ampullary lesions that are amenable to local resection and show no clear evidence of deep invasion or nodal metastasis. Invasive lesions adjacent to the ampulla or lesions requiring lymph node dissection generally fall outside its indications (64, 66, 67).

#### 3.4.2 Efficacy

Available studies suggest that D-LECS can achieve high en bloc and R0 resection rates in selected patients. A Japanese multicenter retrospective study of 206 patients reported an en bloc resection rate of 96.1%, an R0 resection rate of 95.1%, and no recurrence (68). A 2025 single-center study with long-term follow-up further reported no recurrence or stenosis in 18 patients after a median follow-up of 37 months, supporting the acceptability of short-, mid-, and selected longer-term outcomes (69).

In non-ampullary duodenal neuroendocrine tumors, a recent retrospective cohort found that both ESD and LECS achieved very high en bloc resection rates. However, LECS tended to be selected for larger and more deeply invasive tumors, suggesting a possible advantage in avoiding more extensive surgery in carefully chosen cases (70). These results should nevertheless be interpreted cautiously because of potential selection bias and should not be generalized to all duodenal lesions.

#### 3.4.3 Safety

In the duodenum, efficacy must always be interpreted alongside safety. A 2025 systematic review and meta-analysis reported that duodenal ESD was associated with an intraoperative perforation rate of approximately 8.5%, a delayed perforation rate of 2.0%, and a delayed bleeding rate of 3.8% (71). This high-risk profile explains the growing interest in D-LECS. Once perforation or a high-risk resection surface is encountered, immediate laparoscopic repair and reinforcement can reduce the risk of severe delayed complications.

Consistent with this rationale, case series of D-LECS have reported relatively low rates of clinically relevant postoperative adverse events. In addition to retrospective multicenter data, a 2025 prospective study extended the indication to selected periampullary and medial-wall lesions, suggesting that the boundaries of D-LECS may be expanded in highly experienced centers with strict patient selection. Such expansion, however, also increases technical complexity and center dependence (68, 72, 73).

#### 3.4.4 Limitations and directions for improvement

The main limitations of D-LECS are technical complexity and prolonged operative time. Recent studies have reported median operative times ranging from 225 to 303 minutes, with prospective data suggesting an operative time of approximately 296 minutes (63, 64, 72, 73). In addition, duodenal tumors are highly heterogeneous. Tumor size, wall location, distance from the ampulla, circumferential extent, and depth of invasion all influence the optimal operative strategy. Consequently, no single technique is suitable for all cases (64–67).

The most appropriate role of D-LECS is therefore not to replace all endoscopic or surgical treatment for duodenal tumors, but to provide a compromise strategy that combines endoscopic resection with laparoscopic protection for high-risk localized lesions. Wider adoption will depend on clearer risk stratification, improved defect-closure strategies, and structured cross-disciplinary training (64–66).

For duodenal lesions, postoperative management should be guided by final pathology and individualized through multidisciplinary team (MDT) discussion (64, 66). If pathology indicates submucosal invasive adenocarcinoma, a neuroendocrine tumor with high-risk features, lymphovascular invasion, a positive vertical margin, involvement of the periampullary region, or an unacceptable risk of regional lymph node metastasis, additional radical treatment in accordance with oncological principles should be considered (64, 66, 74–76). The choice of subsequent treatment after duodenal LECS should be determined by an MDT, taking into account pathological risk factors, anatomical location, proximity to the ampulla, feasibility of further local or segmental resection, and the patient’s general condition and surgical tolerance. Depending on lesion location and patient condition, this may include segmental duodenal resection, pancreaticoduodenectomy, or other appropriate procedures (74). Conversely, for low-risk adenomas or intramucosal carcinoma with R0 resection, excessive surgery may be avoided under close endoscopic surveillance (66, 69).

The disease-specific evidence reviewed above indicates that LECS has different levels of maturity, risk, and clinical value across gastrointestinal tumor types (17, 69). These differences provide the basis for the following overall assessment of current challenges (17, 69).

## 4 Overall assessment of LECS and current challenges

Across disease types, the principal value of LECS lies not simply in its minimally invasive nature, but in its ability to combine precise resection, organ preservation, and acceptable complication control in selected localized lesions. The strongest evidence currently supports its use in gastric GISTs, complex colorectal polyps, and selected superficial non-ampullary duodenal tumors. In contrast, for epithelial malignancies such as early gastric cancer, LECS remains a highly selective function-preserving strategy rather than a replacement for standard treatment. Taken together, these disease-specific findings indicate that LECS should not be evaluated as a single uniform intervention, but rather as a family of techniques whose value depends on indication, procedure type, and institutional expertise (17, 77, 78).

Several structural limitations should therefore be acknowledged. First, there is substantial heterogeneity in tumor types, procedures, and outcome measures. Studies labeled as “LECS” may include classical LECS, NEWS, CLEAN-NET, local full-thickness resection, D-LECS, or robotic variants, making direct comparison difficult. Second, oncological safety remains the main concern in malignant disease. Exposure-type LECS carries a theoretical risk of digestive fluid contamination and tumor-cell dissemination. Non-exposure techniques may be oncologically safer, but they are more technically demanding, less widely available, and insufficiently validated externally (3, 17, 79).

Third, patient selection bias is a major concern. Many retrospective studies preferentially assign smaller, more superficial, and more favorably located lesions to LECS while reserving higher-risk cases for conventional surgery. This may overestimate the short-term benefits of LECS. The problem is particularly evident in colorectal and duodenal studies, where institutional differences often exist in criteria for additional radical surgery, definitions of R0 resection and local recurrence, and classification of pathologically upstaged cases as failures.

Fourth, the learning curve is substantial, and the center effect is pronounced. LECS depends heavily on advanced endoscopic resection skills, laparoscopic suturing, and intraoperative coordination. Lesions in the duodenum or near the cardia or pylorus demand even greater expertise. Because most available evidence comes from high-volume centers, reproducibility in general practice remains uncertain. Fifth, long-term outcomes and health-economic data remain limited. Although the SENORITA trial suggested benefits in function preservation and quality of life, it did not demonstrate non-inferiority for 3-year disease-free survival. Similarly, although robotic platforms appear potentially cost-effective, they remain far from broadly accessible (21, 29, 62).

A balanced view is therefore that LECS is an important bridge between pure endoscopic treatment and conventional surgery. Its optimal value depends on appropriate patient selection, standardized perioperative management, and acceptance of completion radical resection when required. Shared preoperative decision-making, pathology-guided postoperative management, and long-term follow-up are all essential components of its oncological safety framework (17).

The following subsections further specify these challenges by focusing on three practical dimensions: oncological criteria for treatment escalation, quantification of the learning curve and institutional readiness, and interpretation of the global evidence base.

### 4.1 Oncological criteria for additional radical surgery or treatment escalation

From an oncological perspective, the safety boundaries of LECS should not be defined by a single cross-organ rule. Instead, they should be determined by the integration of final pathological findings, intraoperative exposure risk, and organ-specific oncological principles (18, 20, 45). Accordingly, additional radical surgery or treatment escalation should be considered when LECS reveals any feature associated with an unacceptable risk of residual disease, lymphatic spread, or tumor dissemination. These features include non-R0 resection, particularly a positive or unevaluable vertical margin; pathological invasion beyond the accepted safety range for local resection; lymphatic or vascular invasion; poorly differentiated, undifferentiated, or high-grade malignant components; high-grade tumor budding in colorectal T1 cancer; positive sentinel or regional lymph nodes; intraoperative findings suggesting that the lesion extent exceeds preoperative expectations; and uncontrollable tumor exposure, contamination, or specimen rupture during exposure-based procedures (18, 20, 45, 50).

When these findings are present, LECS should be regarded as a diagnostic, local-control, or bridging procedure rather than definitive oncological treatment. Subsequent management should therefore be individualized through multidisciplinary discussion and guided by the oncological principles relevant to the involved organ. Depending on the clinical context, escalation may include gastrectomy, segmental bowel resection, duodenal resection, or pancreaticoduodenectomy with appropriate lymph node dissection when indicated (6, 17, 44). For GIST, management should be determined by tumor integrity and risk stratification and may include repeat local resection, extended resection, close surveillance, and/or adjuvant targeted therapy (39, 50, 80).

### 4.2 Quantification of the learning curve, structured training, and institutional readiness

At present, no universally accepted case-volume threshold has been established for the learning curve of LECS or its variants (79, 81). Therefore, proficiency should not be assessed solely by operative time, which reflects only one dimension of technical performance. A more appropriate approach is to use composite quality indicators that capture both technical success and clinical safety. These indicators may include en bloc resection rate, R0 resection rate, reliable defect closure rate, intraoperative conversion to open or radical surgery, postoperative leakage, bleeding, delayed perforation, reoperation rate, length of hospital stay, and short-term residual disease or recurrence (2, 6, 79, 82, 83).

The interpretation of these indicators should take into account the heterogeneity of LECS procedures. Because LECS combines advanced endoscopic resection, laparoscopic exposure and suturing, and real-time team coordination, its learning curve varies according to indication, anatomical site, and procedural subtype. Conventional LECS for gastric GIST or selected gastric submucosal tumors may be relatively easier to standardize (2, 77). In contrast, D-LECS, lesions adjacent to the cardia or pylorus, non-exposure LECS for malignant or suspicious lesions, and robotic or third-space variants likely require more advanced team coordination, spatial orientation, and suturing expertise (6, 17, 69).

Structured training should therefore be regarded as a prerequisite for safe dissemination rather than an optional supplement. A practical training pathway may be organized into three progressive levels. The first level focuses on fundamental competencies, including advanced endoscopic resection, endoscopic hemostasis, laparoscopic suturing and ligation, and management of intraoperative complications (82, 83). The second level emphasizes team-based simulation and procedural rehearsal, including ex vivo or animal models, intraoperative communication protocols, specimen retrieval, and defect closure exercises (17, 79, 81). The third level involves mentored clinical practice, performance evaluation, and institutional credentialing (6, 17).

Because direct evidence defining LECS-specific credentialing thresholds remains limited, institutional readiness should be assessed through both individual competence and stable team performance. Such performance can be verified through case logs, complication audits, video-based assessment, and prospective quality registration (17). At the institutional level, LECS should be performed in centers with operative settings that allow intraoperative endoscopy, CO_2_ pneumoperitoneum and insufflation, access to ESD/EFTR instruments, reliable laparoscopic suturing, and conversion surgery when needed. These centers should also have rapid pathological assessment when indicated, an oncological multidisciplinary team, and established perioperative rescue pathways (6, 17, 69).

### 4.3 Global datasets and assessment of evidence strength

From the perspective of the global evidence base, LECS research remains uneven across regions, disease entities, and procedural subtypes (2, 12, 42). Among current indications, the evidence for gastric GIST and selected gastric submucosal tumors is relatively mature. This relative strength is biologically and technically plausible, because these lesions often require complete local resection without routine lymph node dissection, and LECS can help preserve gastric function in anatomically challenging locations (2, 77, 80).

Evidence for complex colorectal lesions is also increasing. Recent systematic reviews and meta-analyses suggest that hybrid laparoscopic-endoscopic approaches may reduce unnecessary segmental resection and improve short-term perioperative outcomes in carefully selected patients (12, 42). For duodenal lesions, current evidence supports feasibility and favorable short-term outcomes in expert centers (6, 69, 84). However, the generalizability of these findings remains limited by small sample sizes, technical complexity, and insufficient multicenter prospective validation (6, 69, 84).

By contrast, direct evidence for LECS in early gastric cancer remains the weakest. Current support is largely derived from small studies of non-exposure local resection and indirect evidence from sentinel node navigation-based function-preserving surgery (17, 22, 26, 85, 86). Therefore, the available evidence supports cautious, indication-specific implementation of LECS according to tumor biology, procedural subtype, center experience, and availability of rescue pathways. At this stage, LECS should not be regarded as a single mature, universally reproducible standard treatment (6, 17).

These limitations define the main directions for future development. Further progress will require improved preoperative risk assessment, more advanced operative platforms, better nodal evaluation, standardized training systems, and prospective multicenter datasets that stratify outcomes by organ, indication, and procedural subtype (6, 12, 17, 42).

## 5 Future directions and prospects

The current limitations of LECS define the priorities for future development. First, artificial intelligence is likely to play an increasing role in preoperative assessment and intraoperative navigation. Existing studies suggest that AI-assisted image analysis has potential for lesion detection, characterization, and assessment of invasion depth in early gastric cancer (87, 88). More broadly, computer vision-based systems for surgical workflow recognition, anatomical annotation, and image-guided navigation are emerging and may enable more precise decision-making and real-time assistance during LECS (89, 90). However, real-time margin assessment, risk prediction, and closed-loop decision-support systems tailored specifically to LECS remain at an early stage.

Second, robotic assistance and novel hybrid devices may further expand the technical capabilities of LECS. Feasibility and comparative studies suggest that robotic platforms can provide more stable visualization, more flexible suturing, and more precise manipulation, particularly in anatomically confined settings that require meticulous defect closure (61, 73, 91). This may be especially relevant for gastric submucosal tumors, lesions near the cardia or pylorus, and selected duodenal lesions. Nevertheless, dual-modality robotic platforms, flexible endoscopic robots, and automated suturing systems remain largely investigational, and their routine role in LECS has not yet been established (61, 73, 91).

Third, sentinel lymph node navigation may offer the most mature path toward truly individualized oncological application of LECS. Randomized and follow-up studies in early gastric cancer have shown that stomach-preserving surgery guided by sentinel node navigation can provide acceptable oncological outcomes together with better quality of life and nutritional status in carefully selected patients (29, 92). As fluorescence tracers, rapid intraoperative pathology, and molecular diagnostic techniques continue to improve, LECS combined with sentinel node navigation may further extend the boundaries of function-preserving treatment in node-negative, low-risk cases.

Fourth, the broad dissemination of LECS requires clearer standards, structured training pathways, and institutional credentialing systems. Recent consensus indicates that LECS and function-preserving strategies combined with sentinel node navigation are gradually moving toward standardization, although coverage in high-level international guidelines remains relatively limited (93). Future training should be advanced across three dimensions: individual technical skills, team collaboration, and case-quality control. Operators should first acquire competence in advanced endoscopic resection, endoscopic hemostasis, laparoscopic suturing, and complication management. They should then progress toward independent practice through simulation training, animal or ex vivo models, mentored instruction, and video-based quality control. Institutional credentialing should emphasize multidisciplinary team decision-making, rapid pathological assessment, the capability to convert to radical surgery, complication rescue pathways, and prospective quality registration. Only when training, certification, and institutional readiness are standardized simultaneously can LECS move from a small number of high-level centers toward more standardized and reproducible clinical application (94).

Taken together, these future directions suggest that the development of LECS will depend not only on technical innovation, but also on better evidence generation, clearer patient selection, and more standardized implementation.

## 6 Conclusion

As a hybrid strategy that combines the precision of endoscopic resection with the operative safety of laparoscopy, LECS has become an important direction in minimally invasive gastrointestinal oncological surgery. In carefully selected patients, it can achieve high en bloc and R0 resection rates, reduce unnecessary organ sacrifice, and promote postoperative recovery.

Current evidence most strongly supports LECS for gastric GISTs, complex colorectal polyps, and selected superficial non-ampullary duodenal tumors. Its application in early gastric cancer should remain limited to highly selected cases combining non-exposure techniques with sentinel lymph node navigation. Such procedures should be performed cautiously, only in experienced centers with clearly defined patient-selection criteria and an established pathway for additional radical surgery when required.

Artificial intelligence, robotic assistance, sentinel lymph node navigation, and standardized training may further advance the field. However, the ultimate clinical role of LECS will depend on higher-quality prospective comparative studies, uniform definitions of indications and outcomes, and continued validation of long-term oncological safety.
